# A multiple‐model generalisation of updating clinical prediction models

**DOI:** 10.1002/sim.7586

**Published:** 2017-12-18

**Authors:** Glen P. Martin, Mamas A. Mamas, Niels Peek, Iain Buchan, Matthew Sperrin

**Affiliations:** ^1^ Farr Institute, Faculty of Biology, Medicine and Health University of Manchester, Manchester Academic Health Science Centre Manchester UK; ^2^ Keele Cardiovascular Research Group Keele University Stoke‐on‐Trent UK; ^3^ NIHR Greater Manchester Primary Care Patient Safety Translational Research Centre University of Manchester Manchester UK; ^4^ Microsoft Research Cambridge UK

**Keywords:** clinical prediction models, logistic regression, model aggregation, model updating, stacked regression, validation

## Abstract

There is growing interest in developing clinical prediction models (CPMs) to aid local healthcare decision‐making. Frequently, these CPMs are developed in isolation across different populations, with repetitive de novo derivation a common modelling strategy. However, this fails to utilise all available information and does not respond to changes in health processes through time and space. Alternatively, model updating techniques have previously been proposed that adjust an existing CPM to suit the new population, but these techniques are restricted to a single model. Therefore, we aimed to develop a generalised method for updating and aggregating multiple CPMs. The proposed “hybrid method” re‐calibrates multiple CPMs using stacked regression while concurrently revising specific covariates using individual participant data (IPD) under a penalised likelihood. The performance of the hybrid method was compared with existing methods in a clinical example of mortality risk prediction after transcatheter aortic valve implantation, and in 2 simulation studies. The simulation studies explored the effect of sample size and between‐population‐heterogeneity on the method, with each representing a situation of having multiple distinct CPMs and 1 set of IPD. When the sample size of the IPD was small, stacked regression and the hybrid method had comparable but highest performance across modelling methods. Conversely, in large IPD samples, development of a new model and the hybrid method gave the highest performance. Hence, the proposed strategy can inform the choice between utilising existing CPMs or developing a model de novo, thereby incorporating IPD, existing research, and prior (clinical) knowledge into the modelling strategy.

## 1 INTRODUCTION

Clinical prediction models (CPMs) aim to predict the presence (diagnostic) or future occurrence (prognostic) of a state or event of interest, and are predominately derived in a single dataset by estimating the associations between the outcome and multiple risk factors (covariates).^1^, ^2^ Such research has proliferated in medical and statistical literature over the past decade. For example, the PROGRESS series detailed a framework of prognostic research themes,^3^, ^4^, ^5^, ^6^ while published guidelines detail CPM development, validation, and impact assessment.^2^, ^7^, ^8^

Notably, post development, the predictive performance of a CPM needs to be evaluated in data samples from populations that are similar (internal validation) and distinct (external validation) to that in which the model was developed.^7^, ^9^ Here, one is interested in the model's ability to separate cases and controls (discrimination), and the agreement between the expected and observed outcome rates across the full risk range (calibration). Although validation studies are rare in practice, they frequently find that the performance of an existing CPM drops when it is applied to observations distinct to those used to derive the model.^5^, ^9^ A common strategy to handle this problem is to develop a new CPM while disregarding existing models.^5^, ^10^, ^11^, ^12^ However, this approach fails to learn from existing CPMs that have been developed for similar outcomes and settings, leads to many CPMs for the same prediction task, and is susceptible to over‐fitting.^12^, ^13^

Alternatively, the prior knowledge encapsulated by an existing CPM can be utilised through model updating techniques, which follow a hierarchical structure to tune an existing CPM to suit the population of interest.^1^, ^10^, ^11^, ^14^ Previous studies have demonstrated the advantages of updating existing CPMs, particularly when only sparse data are available.^10^, ^11^ However, such techniques can only be applied to a single existing CPM, while potentially useful information from other available CPMs is lost. The advantages of combining information across multiple studies through meta‐analysis is widely acknowledged,^15^ with the analogue concept in predictive modelling being model aggregation methods, such as stacked regression.^16^, ^17^, ^18^ Nevertheless, utilising multiple existing CPMs and new data is not fully understood. For instance, it is not clear how the existing CPMs should be selected for aggregation or how new (emerging) risk factors should be added into the aggregate model.^12^ Hence, this study aims to combine model aggregation and model updating to generalise the latter into the multiple‐model setting and formalise the former with respect to model/predictor selection.

This paper considers a situation in which there is a new population with associated data where one is interested in developing a CPM. We will henceforth refer to the data available in the new population as individual participant data (IPD). The paper assumes that the modeller only has access to this one set of IPD and the parameter estimates from multiple previously published CPMs; this contrasts to methods that develop a CPM using multiple sets of IPD by meta‐analysis.^19^, ^20^, ^21^ Thus, the aim of the study is 2‐fold: (1) develop a hybrid method to generalise model updating into the multiple model setting; and (2) study the properties of the method through simulation studies based on synthetic and real‐world data. We illustrate the techniques in a clinical example of 30‐day mortality risk prediction following transcatheter aortic valve implantation (TAVI).

The structure of the paper is as follows. Notation and existing methods are introduced in Section 2. In Section 3, we extend the existing methods into the proposed hybrid method, and Section 4 presents the design and results from a simulation study based on synthetic data. An application of the modelling methods to the TAVI clinical example is described in Section 5, while Section 6 gives the design and results from a simulation study based on TAVI data. Finally, Section 7 discusses the findings of the paper and concludes.

## 2 PRELIMINARIES

Throughout, parameters denoted with a hat accent represent those estimated from the IPD, while parameters without such notation denote those taken as fixed values from previously published research (although these also have an associated uncertainty). Additionally, for ease of exposition, the interpretation of the vector ***X*** (as introduced later) varies according to context. We assume that the outcome of interest is binary and that there are *M* existing logistic regression CPMs, which have been derived for similar outcomes but in distinct populations. The ideas discussed in the paper generalise naturally to models for time‐to‐event outcomes.

The *j*^th^ existing logistic regression CPM (*j* = 1, …, *M*) aims to estimate the probability of a binary outcome occurring, *π*_*j*_(***X***), using a logit‐linear combination of a hypothetical set of covariates, ***X*** = *x*_1_, …, *x*_*P*_, where *P* denotes the number of covariates that are available across all populations (including the IPD). Specifically,

$$\log\left(\frac{\pi_{j}\left(X\right)}{1-\pi_{j}\left(X\right)}\right)=\beta_{0j}+\sum_{p=1}^{P}\beta_{\mathrm{pj}}x_{p},$$

where *β*_*pj*_ denotes the published coefficient for covariate *p* within the *j*^th^ existing CPM; a covariate that is not present in a given CPM simply has coefficient equal to zero. Explicitly, write *S*_*j*_ to be the subset of the *P* covariates that are included in the *j*^th^ existing CPM (ie, the subset of {*p* = 1, …, *P*} such that *p* ∈ *S*_*j*_ if and only if *β*_*pj*_ ≠ 0). Here, we also allow covariates to feature only in the IPD, and not in any of the existing CPMs.

Henceforth, let *j* = *M* + 1 represent the population of interest, with ***X***_*i*,(*M* + 1)_ denoting the vector of *P* covariates for observation *i* = 1, …, *N* in the IPD. Let the *p*^th^ element of ***X***_*i*,(*M* + 1)_ be denoted by *x*_*i*,(*M* + 1),*p*_. Similarly, let *Y*_*i*,(*M* + 1)_ be the corresponding binary outcome. Thus, we explicitly assume that the IPD records all *P* covariates. Consequently, the linear predictor from each existing CPM can be calculated for observations *i* = 1, …, *N* in the IPD using the published coefficients as

$$\log\left(\frac{\pi_{j}\left(X_{i,\left(M+1\right)}\right)\;}{1-\pi_{j}\left(X_{i,\left(M+1\right)}\right)\;}\right)=\beta_{0j}+\sum_{p\in S_{j}}\beta_{\mathrm{pj}}x_{i,\left(M+1\right),p}.$$

Here, *π*_*j*_(***X***_*i*,(*M* + 1)_) represents the estimated event probability, based on existing model *j*, given the covariates for observation *i* within the IPD. To reiterate, the goal is estimating the risk of outcome for a given observation in the IPD using a model tailored for that population—denote this as *π*_(*M* + 1)_(***X***_*i*,(*M* + 1)_).

### 2.1 Individual model updating

Model updating methods have a hierarchical structure to tune a single existing CPM to the population of interest, ranging from logistic re‐calibration to adding new risk factors into the model. We will briefly describe these techniques in this subsection; further details can be found in the literature.^10^, ^11^, ^12^, ^14^ Firstly, model re‐calibration fits a logistic regression model in the IPD, with the linear predictor from exactly one existing CPM as the only covariate. Specifically, given one existing CPM, *j*, logistic re‐calibration is given by modelling

$$\log\left(\frac{\pi_{\left(M+1\right)}\left(X_{i,\left(M+1\right)}\right)}{1-\pi_{\left(M+1\right)}\left(X_{i,\left(M+1\right)}\right)}\right)={\hat{\alpha }}_{0}+{\hat{\alpha }}_{1}\log\left(\frac{\pi_{j}\left(X_{i,\left(M+1\right)}\right)\;}{1-\pi_{j}\left(X_{i,\left(M+1\right)}\right)\;}\right),$$

which can be expanded as

$$\log\left(\frac{\pi_{\left(M+1\right)}\left(X_{i,\left(M+1\right)}\right)}{1-\pi_{\left(M+1\right)}\left(X_{i,\left(M+1\right)}\right)}\right)={\hat{\alpha }}_{0}+{\hat{\alpha }}_{1}\left\{\beta_{0j}+\sum_{p\in S_{j}}\beta_{\mathrm{pj}}x_{i,\left(M+1\right),p}\right\}.$$

The estimated parameters ${\hat{\alpha }}_{0}$ and ${\hat{\alpha }}_{1}$ are called the calibration intercept and slope, respectively; if the existing CPM was perfectly calibrated within the IPD, then ${\hat{\alpha }}_{0}=0$ and ${\hat{\alpha }}_{1}=1$. Conversely, ${\hat{\alpha }}_{0}<0$ implies the *j*^th^ existing CPM systematically over‐predicts risk in the IPD (and *vice versa*), while ${\hat{\alpha }}_{1}<1$ implies the coefficients of the *j*^th^ existing CPM are larger than required within the IPD. Logistic re‐calibration ensures the existing model is well calibrated within the IPD, but it will not change the discrimination because the relative positioning of each observation along the predicted risk range is unaltered.

Hence, to improve the discrimination of a model one can change the relative weightings (prognostic effects) of individual covariates. This can be achieved through model revision, which considers adjustments to parameters of individual covariates after performing logistic re‐calibration. Explicitly, model revision can be expressed as

(1) $$\log\left(\frac{\pi_{\left(M+1\right)}\left(X_{i,\left(M+1\right)}\right)}{1-\pi_{\left(M+1\right)}\left(X_{i,\left(M+1\right)}\right)}\right)={\hat{\alpha }}_{0}+{\hat{\alpha }}_{1}\log\left(\frac{\pi_{j}\left(X_{i,\left(M+1\right)}\right)\;}{1-\pi_{j}\left(X_{i,\left(M+1\right)}\right)\;}\right)+\sum_{p\in S_{j}}{\hat{\delta }}_{p}x_{i,\left(M+1\right),p}.$$

Here, the set of estimated parameters $\left\{{\hat{\delta }}_{p}\;\forall \;p\in S_{j}\right\}$ are obtained using the IPD and represent the alterations of each coefficient after model re‐calibration; hence, the *p*^th^ coefficient following model revision is given by ${\hat{\alpha }}_{1}\beta_{\mathrm{pj}}+{\hat{\delta }}_{p}$. The likelihood ratio test can be used to determine which variables need revision.^11^ Similarly, model extension further considers new terms to be added into the model. This is achieved similarly to Equation (1), except that the final sum is over all *P* covariates, rather than only those in *S*_*j*_.

### 2.2 Model aggregation: stacked regression

Stacked regression weights the linear predictors from the *M* existing CPMs, calculated for each observation in the IPD, in a logit‐linear combination.^16^, ^18^ Hence, stacked regression simultaneously re‐calibrates and combines the existing CPMs by giving an aggregate model of the form

(2) $$\log\left(\frac{\pi_{\left(M+1\right)}\left(X_{i,\left(M+1\right)}\right)}{1-\pi_{\left(M+1\right)}\left(X_{i,\left(M+1\right)}\right)}\right)={\hat{\gamma }}_{0}+\sum_{j=1}^{M}{\hat{\gamma }}_{j}\log\left(\frac{\pi_{j}\left(X_{i,\left(M+1\right)}\right)}{1-\pi_{j}\left(X_{i,\left(M+1\right)}\right)}\right).$$

Only ${\hat{\gamma }}_{0},{\hat{\gamma }}_{1},\ldots ,{\hat{\gamma }}_{M}$ are estimated using the IPD, thereby estimating fewer parameters than alternative model averaging‐based approaches.^16^ Subsequent predictions can be made by either calculating the linear predictors from each existing CPM for the new observation and substituting into Equation (2), or by evaluating the stacked regression model directly where the *p*^th^ coefficient is given by $\sum_{j=1}^{M}{\hat{\gamma }}_{j}\beta_{\mathrm{pj}}$.

Classically, Equation (2) is estimated under the constraint that ${\hat{\gamma }}_{1},\ldots ,{\hat{\gamma }}_{M}\geq 0$ to aid interpretation.^16^ However, this is not strictly required in the context of risk prediction. Within the current paper, we implemented stacked regression both with and without the positivity constraint, and we found that all results were quantitatively similar across both assumptions. Therefore, in the interests of space, we here present only the results without the positivity constraint (the results of stacked regression with the positivity constraint are available on request).

## 3 HYBRID METHOD

While Equation (2) utilises information across multiple CPMs, revisions to covariates within each model, or the addition of new covariates are not considered. One could apply the aforementioned model updating techniques (Equation (1)) before stacked regression, but this would lead to a 2‐step process and potential overfitting. Additionally, it is unknown how the existing CPMs should be selected for stacked regression (with this choice potentially leading to biased parameter estimates), and even a moderate number of existing CPMs could make Equation (2) unstable. Hence, we propose a generalisation of model updating into the multiple‐model setting to address these issues. Specifically, we propose modelling

(3) $$\log\left(\frac{\pi_{\left(M+1\right)}\left(X_{i,\left(M+1\right)}\right)}{1-\pi_{\left(M+1\right)}\left(X_{i,\left(M+1\right)}\right)}\right)={\hat{\beta }}_{0,M+1}+\sum_{j=1}^{M}{\hat{\gamma }}_{j}\log\left(\frac{\pi_{j}\left(X_{i,\left(M+1\right)}\right)\;}{1-\pi_{j}\left(X_{i,\left(M+1\right)}\right)\;}\right)+\sum_{p=1}^{P}{\hat{\beta }}_{p,M+1}x_{i,\left(M+1\right),p}.$$

Hence, the new intercept is given by ${\hat{\beta }}_{0,M+1}+\sum_{j=1}^{M}{\hat{\gamma }}_{j}\beta_{0j}$ and the *p*^th^ coefficient is given by $\sum_{j=1}^{M}{\hat{\gamma }}_{j}\beta_{\mathrm{pj}}+{\hat{\beta }}_{p,M+1}$. Correspondingly, one can recover Equation (1) in the special case of *M* = 1. Importantly, covariates need not feature in every existing CPM and covariates may feature in the final sum that are not in any of the existing CPMs.

To ensure existing CPMs are only revised to an extent supported by the IPD, we propose estimating the parameters by penalised maximum likelihood, where the penalty is equivalent to imposing a prior distribution with heavy tails and a sharp peak at zero. Let $\hat{\theta }=\left({\hat{\gamma }}_{1},{\hat{\gamma }}_{2},\ldots ,{\hat{\gamma }}_{M},{\hat{\beta }}_{1,M+1},\ldots ,{\hat{\beta }}_{P,M+1}\right)$, then parameters were estimated by maximising the following penalised log‐likelihood across all observations *i* = 1, …, *N* in the IPD:

(4) $$\sum_{i=1}^{N}\left\{y_{i}\log\left(\pi_{\left(M+1\right)}\left(X_{i,\left(M+1\right)}\right)\right)+\left(1-y_{i}\right)\log\left(1-\pi_{\left(M+1\right)}\left(X_{i,\left(M+1\right)}\right)\right)\right\}-\lambda \sum_{r=1}^{M+P}\nu_{r}\left|{\hat{\theta }}_{r}\right|.$$

This is, therefore, a lasso regression^22^; consequently, some coefficients can be estimated as zero and if ${\hat{\gamma }}_{j}=0$ then the *j*^th^ existing CPM will be dropped from the model, thereby allowing selection of existing CPMs. The value of *λ* is selected through cross‐validation to minimise the deviance. Additionally, *ν*_*r*_ are chosen prior to modelling to allow differential penalisation across parameters and can be used to incorporate prior (clinical) preference for specific covariates or existing CPMs. In this study, we considered 3 modelling cases (Table 1): (1) set *ν*_*r*_ = 1 ∀ *r* = 1, …, *M* + *P*, (2) set *ν*_1_ = … = *ν*_*M*_ = 0 and *ν*_*M* + 1_ = … = *ν*_*M* + *P*_ = 1, and (3) set $\nu_{r}=\frac{1}{\left|{\hat{\theta }}_{r}^{\text{RIDGE}}\right|}$ for each *r* = 1, …, *M* + *P* where ${\hat{\theta }}_{r}^{\text{RIDGE}}$ is the estimate for parameter *r* obtained by ridge regression. Here, modelling case 1 implies that all parameters receive equal penalty, modelling case 2 implies that only the adjustment parameters $\left({\hat{\beta }}_{1,M+1},\ldots ,{\hat{\beta }}_{P,M+1}\right)$ are penalised, and modelling case 3 implies that parameters with strong covariate‐outcome associations are penalised less than parameters with weaker associations. Modelling case 3 is similar to adaptive lasso, except that the weights are obtained using ridge regression rather than the standard approach of least squares estimation. Within modelling case 3, one first fits Equation (3) using ridge regression to obtain a set of coefficient estimates; Equation (3) is then re‐fit using the inversed‐absolute value of each coefficient as *ν*_*r*_ within Equation (4) (Table 1). We implemented the hybrid method in R version 3.3.1,^23^ using the glmnet package.^24^

**Table 1:** Details of each modelling case considered in the current study, with each altering how the weights (*ν*_*r*_) were pre‐defined when fitting the hybrid method (Equations (3) and (4))

Modelling case	Process to pre‐define the weights (*ν*_*r*_) in Equation (4)
1	Set *ν*_*r*_ = 1 for all parameters in the model—ie, $\nu_{r}=\left\{\begin{array}{ccc} 1 & \text{for} & {\hat{\gamma }}_{1},{\hat{\gamma }}_{2},\ldots ,{\hat{\gamma }}_{M}\\ 1 & \text{for} & {\hat{\beta }}_{1,M+1},\ldots ,{\hat{\beta }}_{P,M+1} \end{array}\right.$
2	Set *ν*_*r*_ = 1 for any parameter representing an adjustment to individual covariates, and set *ν*_*r*_ = 0 for all weights of existing CPMs—ie, $\nu_{r}=\left\{\begin{array}{ccc} 0 & \text{for} & {\hat{\gamma }}_{1},{\hat{\gamma }}_{2},\ldots ,{\hat{\gamma }}_{M}\\ 1 & \text{for} & {\hat{\beta }}_{1,M+1},\ldots ,{\hat{\beta }}_{P,M+1} \end{array}\right.$
3	Perform the following steps: Fit Equation (3) using ridge regression Store the estimates of the coefficients—call these ${\hat{\theta }}^{\text{RIDGE}}=\left({\hat{\gamma }}_{1}^{\text{RIDGE}},\ldots ,{\hat{\gamma }}_{M}^{\text{RIDGE}},{\hat{\beta }}_{1,M+1}^{\text{RIDGE}},\ldots ,{\hat{\beta }}_{P,M+1}^{\text{RIDGE}}\right)$ Fit Equation (3) again using the likelihood in Equation (4), and set each *ν*_*r*_ to the inversed‐absolute value of the corresponding ${\hat{\theta }}^{\text{RIDGE}}$—ie, $\nu_{r}=\frac{1}{\left|{\hat{\theta }}_{r}^{\text{RIDGE}}\right|}$

## 4 SYNTHETIC SIMULATION STUDY

### 4.1 Simulation design

Details of the simulation procedure are given in Supporting Information A and follow a similar approach to previous simulation studies.^13^ In summary, we generated data for 6 populations, with each including 50 covariates that were simulated as a mixture of continuous and binary variables. The covariates were generated within 10 clusters of serially correlated variables to mimic multiple risk factors that measure similar characteristics. Five of the populations (each of size 5000) represented those previously used to derive a CPM. Thus, *M* = 5 existing CPMs were derived in distinct and potentially heterogeneous populations, with each including a potentially overlapping subset of the 50 simulated covariates (resampled within each iteration). The sixth population acted as the IPD on which one is interested in deriving a new model. The size of the IPD was varied through 200, 300, 500, 1000, 2000, and 5000 observations and was used to apply model updating, stacked regression, and the hybrid method. Additionally, a new model was derived in the IPD using backwards selection with Akaike Information Criterion (AIC) and by ridge regression. For all modelling strategies, the covariates that were available in the IPD were restricted to be exactly those that were included across the 5 existing CPMs.

Binary responses (mean event rate of 25%) were generated from a population‐specific generating logistic regression model, the coefficients of which were assumed (without loss of generality) to be those at the “start” of each cluster of serially correlated variables. Predictor effect heterogeneity between the populations was induced by applying N(0, *σ*^2^) variation to the coefficients of the population‐specific generating logistic regression model (see Martin et al^13^ and Supporting Information A for details). Higher values of *σ* induce greater differences in covariate‐outcome associations across the populations; we varied *σ* through 0, 0.125, 0.25, 0.375, 0.5, and 0.75.

The performance of the 5 existing CPMs, the 5 updated existing CPMs, stacked regression, the hybrid method, and model re‐development was assessed in a further independent sample (of size 5000) drawn from the same distribution as the simulated IPD. This represents a validation study using independent samples from the same underlying population as that for model derivation. Performance was assessed in terms of mean square error in the predicted risks, calibration, and discrimination. Calibration was quantified with a calibration intercept and slope, with reference values of zero and one, respectively.^25^ Discrimination was quantified with the area under the receiver operating characteristic curve (AUC).

All simulation scenarios (ie, each combination of IPD sample size and σ) were repeated over 1000 iterations, with mean performance estimates and empirical standard errors calculated. The R code is available in the online Supporting Information.

### 4.2 Simulation results

**Figure 1:**
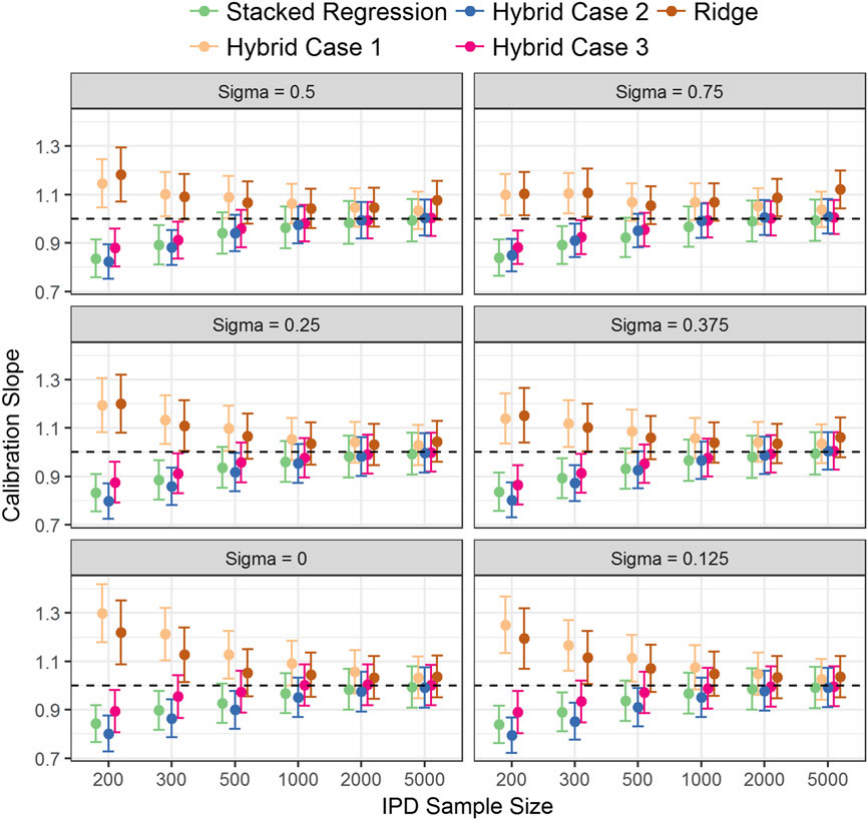
Calibration slope of stacked regression, the hybrid models, and ridge regression for the synthetic simulation study across all between‐population heterogeneity (*σ*) and individual participant data (IPD) sample sizes. Results for the individual model updating and the re‐development by AIC selection have been removed from the plot for clarity [Colour figure can be viewed at wileyonlinelibrary.com]

The hybrid method consistently outperformed individual model revision in terms of calibration, discrimination, and mean square error (Figure 1, Table 2 and Supporting Information A: **Table A1**). This highlights the advantage of incorporating evidence from multiple CPMs while concurrently revising individual parameters using the IPD. Thus, the hybrid method (across the 3 modelling cases) resulted in the lowest mean square error in the predicted risks of all modelling strategies (Supporting Information A: **Table A1**).

**Table 2:** AUC (standard error) results for the synthetic simulation study. Bold items indicate the maximum AUC in each combination of IPD sample size and value of σ. For clarity, results for IPD sample sizes of 300 and 2000 are given in Supporting Information A: Table A2

Model	IPD Sample Size	σ = 0.000	σ = 0.125	σ = 0.250	σ = 0.375	σ = 0.500	σ = 0.750
Model revision^a^	200	0.677 (0.009)	0.673 (0.009)	0.674 (0.009)	0.680 (0.009)	0.691 (0.008)	0.722 (0.008)
Stacked regression	200	**0.712 (0.008)**	**0.710 (0.008)**	0.709 (0.008)	0.708 (0.008)	0.714 (0.008)	0.728 (0.008)
Hybrid case 1	200	0.707 (0.008)	0.707 (0.008)	0.711 (0.008)	**0.720 (0.008)**	**0.735 (0.008)**	**0.769 (0.007)**
Hybrid case 2	200	0.709 (0.008)	0.708 (0.008)	**0.713 (0.008)**	**0.720 (0.008)**	0.733 (0.008)	0.766 (0.008)
Hybrid case 3	200	0.697 (0.008)	0.697 (0.008)	0.704 (0.008)	0.712 (0.008)	0.728 (0.008)	0.764 (0.008)
Ridge regression	200	0.681 (0.009)	0.686 (0.009)	0.698 (0.008)	0.709 (0.008)	0.725 (0.008)	0.760 (0.008)
Model revision^a^	500	0.684 (0.009)	0.682 (0.009)	0.689 (0.009)	0.698 (0.008)	0.712 (0.008)	0.740 (0.008)
Stacked regression	500	0.721 (0.008)	0.719 (0.008)	0.718 (0.008)	0.721 (0.008)	0.723 (0.008)	0.735 (0.008)
Hybrid case 1	500	**0.722 (0.008)**	**0.723 (0.008)**	**0.732 (0.008)**	**0.745 (0.008)**	**0.757 (0.008)**	**0.790 (0.007)**
Hybrid case 2	500	0.721 (0.008)	0.722 (0.008)	0.730 (0.008)	0.742 (0.008)	0.754 (0.008)	0.788 (0.007)
Hybrid case 3	500	0.719 (0.008)	0.720 (0.008)	0.729 (0.008)	0.742 (0.008)	0.754 (0.008)	0.788 (0.007)
Ridge regression	500	0.708 (0.008)	0.713 (0.008)	0.724 (0.008)	0.738 (0.008)	0.750 (0.008)	0.784 (0.007)
Model revision^a^	1000	0.688 (0.009)	0.687 (0.009)	0.694 (0.009)	0.707 (0.008)	0.719 (0.008)	0.748 (0.008)
Stacked regression	1000	0.726 (0.008)	0.722 (0.008)	0.721 (0.008)	0.724 (0.008)	0.724 (0.008)	0.742 (0.008)
Hybrid case 1	1000	**0.729 (0.008)**	**0.729 (0.008)**	**0.738 (0.008)**	**0.752 (0.008)**	**0.764 (0.008)**	**0.798 (0.007)**
Hybrid case 2	1000	0.728 (0.008)	0.728 (0.008)	0.736 (0.008)	0.751 (0.008)	0.763 (0.008)	0.796 (0.007)
Hybrid case 3	1000	0.728 (0.008)	0.728 (0.008)	0.737 (0.008)	0.751 (0.008)	0.763 (0.008)	0.797 (0.007)
Ridge regression	1000	0.722 (0.008)	0.724 (0.008)	0.733 (0.008)	0.748 (0.008)	0.760 (0.008)	0.794 (0.007)
Model revision^a^	5000	0.689 (0.009)	0.692 (0.009)	0.701 (0.008)	0.713 (0.008)	0.73 (0.008)	0.755 (0.008)
Stacked regression	5000	0.728 (0.008)	0.725 (0.008)	0.724 (0.008)	0.727 (0.008)	0.732 (0.008)	0.743 (0.008)
Hybrid case 1	5000	**0.734 (0.008)**	**0.736 (0.008)**	**0.745 (0.008)**	**0.761 (0.008)**	**0.776 (0.007)**	**0.805 (0.007)**
Hybrid case 2	5000	**0.734 (0.008)**	**0.736 (0.008)**	**0.745 (0.008)**	0.760 (0.008)	**0.776 (0.007)**	0.804 (0.007)
Hybrid case 3	5000	**0.734 (0.008)**	**0.736 (0.008)**	**0.745 (0.008)**	**0.761 (0.008)**	**0.776 (0.007)**	**0.805 (0.007)**
Ridge regression	5000	0.733 (0.008)	0.735 (0.008)	0.744 (0.008)	0.759 (0.008)	0.775 (0.007)	0.803 (0.007)

a *Results of model revision are from one of the simulated existing CPMs, with results being quantitatively similar across all 5 simulated existing CPMs*.

The calibration slope of the ridge regression model was significantly different from one for IPD of smaller than 500 observations (Figure 1). By contrast, the hybrid methods were well calibrated when the IPD had over 200 observations. Modelling case 1 of the hybrid method (that penalised all parameters equally) was susceptible to over‐shrinkage of the parameter estimates, particularly at low IPD sample sizes where the calibration slope was significantly above one.

**Figure 2:**
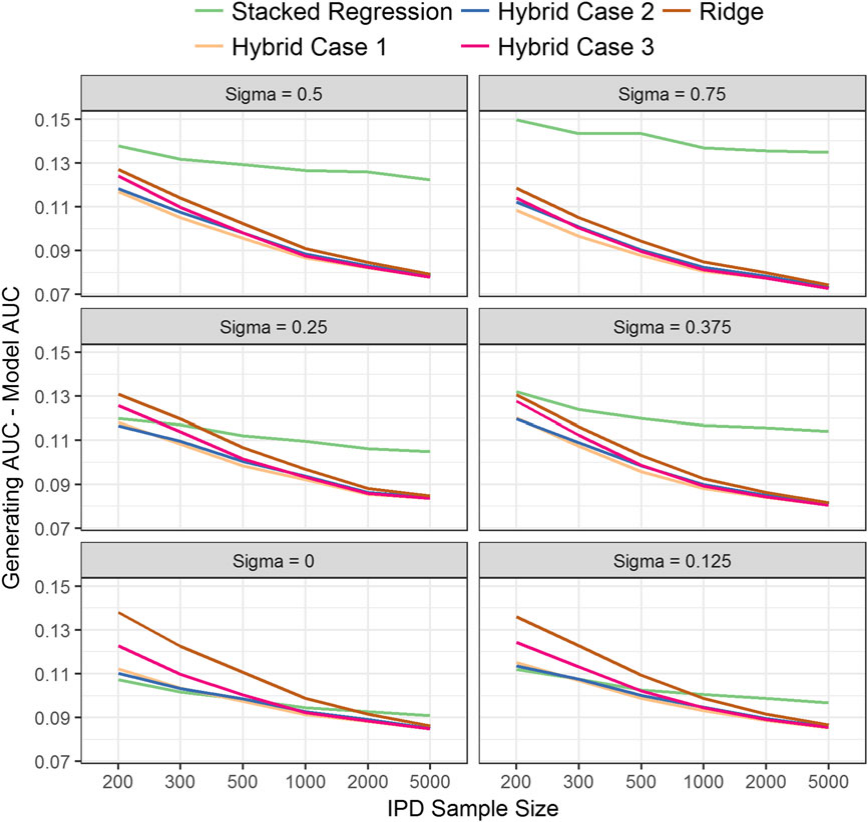
Difference between the generating model AUC and the AUC of each modelling method for the synthetic simulation study across all between‐population heterogeneity (*σ*) and individual participant data (IPD) sample sizes. Results for the individual model updating and the re‐development by AIC selection have been removed from the plot for clarity [Colour figure can be viewed at wileyonlinelibrary.com]

Regarding discrimination, individual model revision had higher AUC than stacked regression in situations of large predictor‐effect heterogeneity (σ > 0.5) and large IPD sample sizes (*n* > 1000) (Table 2). When the IPD had 200 or 300 observations, and there was low predictor effect heterogeneity across populations (*σ* < 0.125), stacked regression and the hybrid methods had similar AUC values, with both being higher than the re‐development methods. In the reverse situation of large IPD samples, ridge regression and the hybrid method had similar AUC values, with these being significantly higher than stacked regression. As the between‐population‐heterogeneity (*σ*) increased, the absolute difference between the AUC of the data generating model and the AUC of the stacked regression model increased, but this was not observed for the hybrid method (Figure 2). This is expected because the hybrid method allows the revision of individual parameters when prognostic effects in the population of interest are markedly different to the existing CPMs.

## 5 APPLICATION TO TAVI RISK PREDICTION

Aortic stenosis is a common heart valve disease in Europe and North America, largely caused by an age‐related degeneration and calcification. TAVI is a non‐invasive and efficacious treatment option for patients with aortic stenosis who are deemed high‐operative risk.^26^, ^27^ Consequently, assessment of a patient's procedural risk forms an important part of the decision‐making process for treatment of aortic stenosis. Currently available CPMs for predicting 30‐day mortality risk post TAVI are scarce, but 4 existing models were considered in this study; namely, the German Aortic Valve model,^28^ the FRANCE‐2 model,^29^ the Italian OBSERVANT model,^30^ and the American College of Cardiology model.^31^ A summary of the covariates and corresponding coefficients of each existing TAVI‐CPM are given in Table 3. Notably, while each model shares similar risk factors, the definitions can vary between models (eg, age bands) and some risk factors are only included in a subset of the models (eg, gender). However, stacked regression and the hybrid method allow the existing CPMs to have varying sets of risk factors, unlike alternative model aggregation methods.^17^

**Table 3:** Coefficients from each of the previously published TAVI models

Covariate	German AV	FRANCE‐2	OBSERVANT	ACC	Coefficient Difference^a^
Age 66–70	0.461	‐	‐	‐	0.461
Age 71–75	0.909	‐	‐	‐	0.909
Age 76–80	1.292	‐	‐	‐	1.292
Age 81–85	1.782	‐	‐	‐	1.782
Age > 85	2.351	‐	‐	‐	2.351
Age ≥ 90	‐	0.420	‐	‐	0.420
Age per 5 years	‐	‐	‐	0.122	0.122
Female	0.357	‐	‐	‐	0.357
BMI <22 kg/m^2^	0.359	‐	‐	‐	0.359
BMI <18.5 kg/m^2^	‐	0.820	‐	‐	0.820
BMI 18.5–29.9 kg/m^2^	‐	0.410	‐	‐	0.410
BMI >35 kg/m^2^	0.393	‐	‐	‐	0.393
NYHA class IV	0.532	0.580	0.600	0.223	0.377
MI within 3 weeks	0.825	‐	‐	‐	0.825
Critical pre‐op	0.662	0.870	0.750	‐	0.870
Pulmonary hypertension	0.398	0.370	0.600	‐	0.600
No sinus rhythm	0.343	‐	‐	‐	0.343
LVEF 30–50%	0.283	‐	‐	‐	0.283
LVEF <30%	0.570	‐	‐	‐	0.570
LVEF <40%	‐	‐	0.450	‐	0.450
Prior cardiac surgery	0.307	‐	‐	‐	0.307
Arterial vessel disease	0.359	‐	‐	‐	0.359
COPD	0.318	0.500	‐	0.511	0.511
Dialysis	1.164	1.060	‐	1.179	1.179
Emergency	1.057	‐	‐	‐	1.057
Non‐TF access	‐	‐	‐	0.673	0.673
TA access	‐	0.700	‐	‐	0.700
Other access	‐	0.780	‐	‐	0.780
eGFR <45 mL/min	‐	‐	0.900	‐	0.900
eGFR per 5 units	‐	‐	‐	−0.069	0.069
Diabetes	‐	‐	0.600	‐	0.600
Prior BAV	‐	‐	0.450	‐	0.450
Acuity category 2^b^	‐	‐	‐	0.451	0.451
Acuity category 3^b^	‐	‐	‐	0.993	0.993
Acuity category 4^b^	‐	‐	‐	1.207	1.207

a *The difference in coefficient value for each covariate across the 4 TAVI‐CPMs (ie, the maximum coefficient value minus the minimum coefficient value for each variable)*.

b *Defined as a composite of procedure urgency, pre‐procedure shock, inotropes, mechanical assist device, or cardiac arrest*.^31^

*Abbreviations: ACC, American College of Cardiology model; BAV, balloon aortic valvuloplasty; BMI, body mass index; COPD, chronic obstructive pulmonary disease; eGFR, estimated glomerular filtration rate; German AV, German Aortic Valve model; LVEF, left ventricular ejection fraction; MI, myocardial infarction; TF, transfemoral; TA, transapical*.

The UK TAVI registry was used as the IPD, which included all 6339 patients who underwent TAVI between 2009 and 2014 across the 32 TAVI centres in England and Wales.^32^ Model updating, stacked regression, and the hybrid method were applied to the existing TAVI‐CPMs in the UK TAVI registry and a new model was derived by AIC backwards selection and by ridge regression. Exactly those covariates that were included in the existing TAVI‐CPMs were considered when applying each of the modelling techniques; a sensitivity analysis was undertaken that removed this restriction. Specifically, frailty is thought to be predictive of mortality after TAVI.^33^ Two measures of frailty were available in the UK TAVI registry, which were not included in any existing TAVI‐CPM; namely, the KATZ index of activities of daily living score^34^ and the Canadian Study of Health and Aging frailty scale.^35^ Therefore, the sensitivity analysis allowed these frailty measures to be considered within the modelling techniques.

Predictive performance of all models was assessed in terms of calibration and discrimination, with all models validated using bootstrapping with 100 replications to correct for in‐sample optimism.^1^, ^36^ All missing data within the UK TAVI registry were imputed using multiple imputation, with 10 imputed datasets generated.^37^ The endpoint of 30‐day mortality was included in the imputation models of missing covariates.^38^ Note that the purpose here was not to provide a validation of the TAVI‐CPMs in the UK TAVI registry, neither was it to develop a new CPM for UK TAVI patients; rather, the aim was to illustrate and compare the proposed method in a real‐world clinical example.

### 5.1 TAVI application results

The mean 30‐day mortality rate observed in the UK TAVI registry was 5.14%. While the original TAVI‐CPMs were miscalibrated and had low discrimination when applied in the UK registry, model re‐calibration resulted in well‐calibrated models both before and after bootstrap correction (Table 4). The increase in AUC between model re‐calibration and model revision was marginal because few of the parameters were adjusted after re‐calibration. Additionally, the discrimination of the stacked regression model was similar to that of the individual TAVI‐CPMs because the majority of the weighting was applied to the German Aortic Valve model, the FRANCE‐2 model and the American College of Cardiology model, thus resulting in similar coefficient values across the revised and stacked regression models (Supporting Information B: **Table B1**). The discrimination of the hybrid method was indistinguishable across modelling cases 1, 2, and 3, with each having higher AUCs than those obtained by individual model revision (Table 4). The hybrid and re‐development approaches shared similar predictive performance and coefficient estimates were similar across stacked regression, hybrid, and re‐development (**Table B1**).

**Table 4:** Performance measures before (apparent) and after bootstrap corrected optimism when modelling in the whole TAVI dataset. Note that no correction is needed when validating the original models because no new parameters are estimated. A calibration intercept and slope of zero and one, respectively, would indicate a well‐calibrated model

	Calibration Intercept (95% CI)		Calibration Slope (95% CI)		AUC (95% CI)	
Model	Apparent	Bootstrap	Apparent	Bootstrap	Apparent	Bootstrap
Original CPMs						
German AV	−0.41 (−0.53, −0.30)	N/A	0.48 (0.35, 0.61)	N/A	0.60 (0.57, 0.64)	N/A
FRANCE‐2	−0.65 (−0.76, −0.54)	N/A	0.71 (0.53, 0.88)	N/A	0.63 (0.60, 0.66)	N/A
OBSERVANT	−0.36 (−0.47, −0.24)	N/A	0.35 (0.21, 0.50)	N/A	0.56 (0.53, 0.59)	N/A
ACC	−0.01 (−0.12, 0.10)	N/A	0.69 (0.53, 0.85)	N/A	0.64 (0.61, 0.67)	N/A
Model recalibration						
German AV	0.00 (−0.11, 0.11)	0.00 (−0.11, 0.11)	1.00 (0.73, 1.27)	1.02 (0.75, 1.29)	0.60 (0.57, 0.64)	0.60 (0.57, 0.64)
FRANCE‐2	0.00 (−0.11, 0.11)	0.00 (−0.11, 0.11)	1.00 (0.76, 1.24)	1.02 (0.78, 1.26)	0.63 (0.60, 0.66)	0.63 (0.60, 0.66)
OBSERVANT	0.00 (−0.11, 0.11)	0.00 (−0.11, 0.11)	1.00 (0.60, 1.40)	1.07 (0.66, 1.47)	0.56 (0.53, 0.59)	0.56 (0.53, 0.60)
ACC	0.00 (−0.11, 0.11)	0.00 (−0.11, 0.11)	1.00 (0.77, 1.23)	1.02 (0.79, 1.25)	0.64 (0.61, 0.67)	0.64 (0.61, 0.67)
Model revision						
German AV	0.00 (−0.11, 0.11)	0.00 (−0.11, 0.11)	1.00 (0.78, 1.22)	0.87 (0.65, 1.10)	0.63 (0.59, 0.66)	0.61 (0.58, 0.64)
FRANCE‐2	0.00 (−0.11, 0.11)	0.00 (−0.11, 0.11)	1.00 (0.79, 1.21)	0.94 (0.72, 1.15)	0.64 (0.61, 0.67)	0.63 (0.60, 0.66)
OBSERVANT	0.00 (−0.11, 0.11)	0.00 (−0.11, 0.11)	1.00 (0.69, 1.31)	0.93 (0.62, 1.24)	0.59 (0.55, 0.62)	0.58 (0.54, 0.61)
ACC	0.00 (−0.11, 0.11)	0.00 (−0.11, 0.11)	1.00 (0.79, 1.21)	0.95 (0.73, 1.16)	0.64 (0.61, 0.67)	0.64 (0.60, 0.67)
Stacked regression	0.00 (−0.11, 0.11)	0.00 (−0.11, 0.11)	1.00 (0.79, 1.21)	0.98 (0.77, 1.19)	0.64 (0.61, 0.68)	0.64 (0.61, 0.67)
Hybrid method						
Case 1	0.00 (−0.11, 0.11)	0.00 (−0.11, 0.11)	1.24 (0.96, 1.53)	1.08 (0.80, 1.36)	0.67 (0.64, 0.71)	0.64 (0.61, 0.68)
Case 2	0.00 (−0.11, 0.11)	0.00 (−0.11, 0.11)	1.09 (0.89, 1.28)	0.93 (0.74, 1.13)	0.67 (0.63, 0.70)	0.64 (0.61, 0.67)
Case 3	0.00 (−0.11, 0.11)	0.00 (−0.11, 0.11)	1.13 (0.93, 1.32)	0.96 (0.77, 1.15)	0.67 (0.64, 0.71)	0.65 (0.61, 0.68)
CPM re‐development						
AIC	0.00 (−0.11, 0.11)	0.00 (−0.11, 0.11)	1.00 (0.84, 1.16)	0.81 (0.64, 0.97)	0.68 (0.65, 0.71)	0.65 (0.62, 0.68)
Ridge regression	0.00 (−0.11, 0.11)	0.00 (−0.11, 0.11)	1.29 (1.08, 1.51)	1.13 (0.91, 1.34)	0.68 (0.65, 0.71)	0.66 (0.63, 0.69)

*Abbreviations: ACC, American College of Cardiology model; German AV, German Aortic Valve model*.

Interestingly, the mean difference in the coefficients across the 4 TAVI‐CPMs given in Table 3 was 0.692, with a lower and upper quantile of 0.385 and 0.885, respectively. Such differences can be compared with those generated across values of *σ* from the synthetic simulation study in Section 4. Specifically, when *σ* = 0.25 the mean difference in coefficients generated across populations was 0.63 and that for *σ* = 0.375 was 0.95. Hence, one can quantitatively compare the results from the synthetic simulation with those using the real‐world data.

The sensitivity analysis that considered the addition of new covariates into the modelling demonstrated that both KATZ and Canadian Study of Health and Aging frailty scores were added during individual model extension, the hybrid method, and re‐development (Supporting Information B: **Table B2**). Moreover, the addition of such frailty measures resulted in an increase in the AUC from those given in the main analysis (Table 5). Because stacked regression does not consider new parameters, the sensitivity analysis results for this method are identical to the main analysis, which demonstrates an advantage of the proposed hybrid method.

**Table 5:** Performance measures before (apparent) and after bootstrap corrected optimism when modelling in the whole TAVI dataset in the sensitivity analysis that considered the addition of frailty (KATZ and Canadian Study of Health and Aging) into the models

	Calibration Intercept (95% CI)		Calibration Slope (95% CI)		AUC (95% CI)	
Model	Apparent	Bootstrap	Apparent	Bootstrap	Apparent	Bootstrap
Model extension						
German AV	0.00 (−0.11, 0.11)	0.00 (−0.11, 0.11)	1.00 (0.81, 1.19)	0.88 (0.69, 1.08)	0.65 (0.61, 0.70)	0.64 (0.59, 0.68)
FRANCE‐2	0.00 (−0.11, 0.11)	0.00 (−0.11, 0.11)	1.00 (0.82, 1.18)	0.93 (0.75, 1.12)	0.67 (0.63, 0.71)	0.66 (0.62, 0.70)
OBSERVANT	0.00 (−0.11, 0.11)	0.00 (−0.11, 0.11)	1.00 (0.77, 1.23)	0.93 (0.70, 1.16)	0.64 (0.59, 0.68)	0.63 (0.58, 0.68)
ACC	0.00 (−0.11, 0.11)	0.00 (−0.11, 0.11)	1.00 (0.82, 1.18)	0.94 (0.76, 1.13)	0.67 (0.63, 0.71)	0.66 (0.62, 0.70)
Stacked regression	0.00 (−0.11, 0.11)	0.00 (−0.11, 0.11)	1.00 (0.79, 1.21)	0.98 (0.77, 1.19)	0.64 (0.61, 0.68)	0.64 (0.61, 0.67)
Hybrid method						
Case 1	0.00 (−0.11, 0.11)	0.00 (−0.11, 0.11)	1.23 (0.96, 1.49)	1.07 (0.80, 1.34)	0.69 (0.65, 0.73)	0.66 (0.62, 0.70)
Case 2	0.00 (−0.11, 0.11)	0.00 (−0.11, 0.11)	1.12 (0.94, 1.30)	0.97 (0.79, 1.15)	0.69 (0.65, 0.72)	0.66 (0.62, 0.70)
Case 3	0.00 (−0.11, 0.11)	0.00 (−0.11, 0.11)	1.10 (0.92, 1.27)	0.94 (0.77, 1.12)	0.69 (0.66, 0.73)	0.67 (0.63, 0.70)
CPM re‐development						
AIC	0.00 (−0.11, 0.11)	0.00 (−0.11, 0.11)	1.00 (0.85, 1.15)	0.82 (0.67, 0.98)	0.70 (0.66, 0.73)	0.66 (0.63, 0.70)
Ridge regression	0.00 (−0.11, 0.11)	0.00 (−0.11, 0.11)	1.27 (1.07, 1.47)	1.11 (0.91, 1.30)	0.70 (0.66, 0.73)	0.67 (0.64, 0.71)

*Abbreviations: ACC, American College of Cardiology model; German AV, German Aortic Valve model*.

## 6 EMPIRICAL SIMULATION STUDY

A simulation based on the TAVI dataset was undertaken where samples of 200, 500, and 1000 observations were randomly extracted (without replacement) from the UK TAVI registry. Such “development cohorts” aimed to represent the situation of developing a CPM to help inform local healthcare decisions where limited data will be available. In each development cohort, model updating, stacked regression, and the hybrid method were applied to the 4 TAVI‐CPMs; new CPMs were derived using AIC backwards selection and ridge regression. Those patients who were not sampled into a development cohort were used to validate the models; hence, the size of the validation sample was 6139, 5839, and 5339 for development sample sizes of 200, 500, and 1000, respectively.

The observed 30‐day mortality rate in the TAVI registry (5.14%) was insufficient to accurately re‐develop a logistic regression model in IPD of sizes 200, 500, and 1000.^36^ Therefore, we used the observed covariate data from the UK TAVI registry to generate binary events with an overall event rate of 25%. Binary endpoints were simulated for each patient in the TAVI registry (*i* = 1, …, 6339) by assuming that *P*(*Y*_*i*_ = 1) = *q*_*i*_ with

$$\log\left(\frac{q_{i}}{1-q_{i}}\right)=\beta_{0}+\left(\sum_{p=1}^{P}\left\{\left(\frac{1}{4}\sum_{j=1}^{M=4}\beta_{p,j}\right)+\epsilon_{p}\right\}x_{i,p}\right)+\left(\sum_{c=1}^{4}{\tilde{\beta }}_{c}{\tilde{x}}_{i,c}\;\right)$$

where $\epsilon_{p}∼N\left(0,\sigma_{p}^{2}\right)$, with $\sigma_{p}^{2}∼\text{Uniform}\left(0,0.75\right)$, and where *β*_*p*, *j*_ denotes the published coefficient from the *j*^*th*^ TAVI‐CPM for covariate *p* (Table 3). Additionally, we generated 4 binary covariates, ${\tilde{x}}_{i,c}$, with corresponding coefficients $\tilde{\beta }∼\text{Uniform}\left(1.4,\;1.6\right)$ and success probability ranging from 30% to 40%, each representing unmeasured covariates, which were not available for inclusion in any modelling strategy. We restricted $\left(\frac{1}{4}\sum_{j=1}^{M=4}\beta_{p,j}+\epsilon_{p}\right)$ to have the same sign as the corresponding $\frac{1}{4}\sum_{j=1}^{M=4}\beta_{p,j}$ and set $\left(\frac{1}{4}\sum_{j=1}^{M=4}\beta_{p,j}+\epsilon_{p}\right)=0$ for any *p* ∈ [1, *P*] that failed this condition (ie, non‐opposing effects between the TAVI‐CPMs and the IPD). For each of the development cohort sizes (200, 500, or 1000), the simulation was repeated 100 times in each of the 10‐multiple imputed TAVI datasets, resulting in 1000 total replications. The simulations were implemented using R, and the code is available in the online Supporting Information.

**Figure 3:**
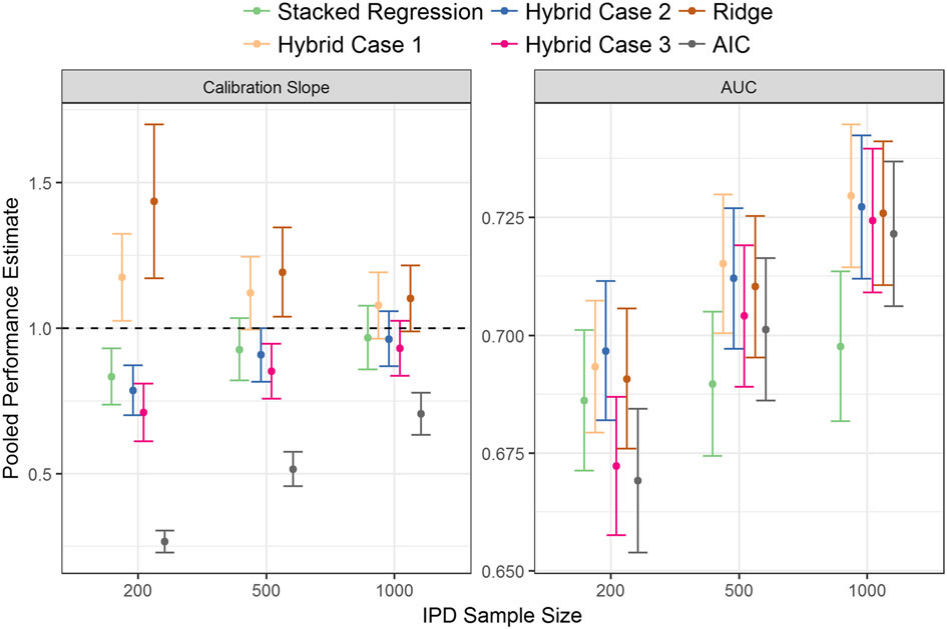
Calibration slope and AUC values for stacked regression, the hybrid method (modelling cases 1, 2, and 3), and re‐development from the TAVI simulation across all individual participant data (IPD) sample sizes [Colour figure can be viewed at wileyonlinelibrary.com]

The results of the empirical simulation are depicted in Figure 3. The calibration slope of the AIC re‐developed model was significantly below one for all development cohort sizes, which indicates overfitting; the calibration slope for ridge regression was significantly higher than one due to slight over‐shrinkage, particularly at the smaller sample sizes. Stacked regression and the hybrid method were well calibrated for development sizes of 500 and 1000. For development cohorts of size 200 and 500 observations, the AUC of all methods were comparable, with the hybrid method under modelling cases 1 and 2 having numerically highest discrimination. For development cohorts sample sizes of 1000 observations, the AUC of the hybrid method was significantly higher than stacked regression and individual model revision (model revision results not shown for clarity).

## 7 DISCUSSION

This study has presented a novel method to generalise model updating techniques to situations where multiple existing models, each with potentially varying sets of covariates, are available. The predictive performance of the hybrid method was contrasted with that of current approaches. This study confirms previous work in finding that it can be advantageous to incorporate existing models when deriving a new CPM, particularly given relatively small samples sizes.^11^, ^13^, ^16^, ^17^, ^39^ The methods that utilised multiple existing CPMs (stacked regression or hybrid) outperformed individual model revision in the majority of circumstances. Although the differences in predictive performance of each method were subtle, one would expect this because the likelihood of the hybrid method converges to stacked regression or re‐development in small or large IPD samples, respectively. Thus, the hybrid method proposed here can assist in optimising the choice between model aggregation and model re‐development.^13^ By generalising the model updating techniques into the multiple‐model setting, one can directly incorporate previous research and models into the modelling strategy.

Previous work in the area of combining IPD with model aggregation has relied on the stringent assumption that all existing CPMs share a common set of risk factors.^17^ While model updating and stacked regression techniques relax this assumption, each has their own inherent restrictions. For instance, model updating has previously been limited to adapting one existing CPM, and in the stacked regression literature it has not previously been discussed how new covariates can be added into the resultant meta‐model.^12^, ^16^ For this reason, the hybrid method presented here allows individual parameters to be revised during model aggregation, but only to the extent supported by the IPD. Revisions to any aggregated parameters will be small (large) if the existing CPMs perform well (poorly) in the IPD, but the use of *L*_1_ penalisation to estimate the unknown parameters means that relatively strong evidence will be required for any such revisions. Additionally, unlike stacked regression, the hybrid method provides a pragmatic way of considering the addition of new covariates into the model aggregation (eg, frailty variables in the TAVI example: Supporting Information B).

By allowing differential penalisation across the existing models (Equation (4)), one can directly incorporate prior knowledge into the modelling strategy. For instance, an existing CPM could be penalised less if several external validation studies have shown said CPM to generalise well, or if expert knowledge leads us to believe, a priori, that a CPM should suit the population of interest. Alternatively, the degree of penalisation could be based on the size of data used to initially derive the existing CPMs. Further work is needed regarding the translation between prior knowledge and a quantifiable weighting of the penalisation (ie, how to pre‐define *ν*_*r*_). For instance, one could alter our modelling case 2 so that all existing models are penalised by the same constant, which is potentially different to the penalty applied across all adjustment parameters, with such weights selected through cross‐validation. However, such cross‐validation approaches to define *ν*_*r*_ will be computationally demanding. Modelling case 2, where only the adjustment terms were penalised, will likely be sufficient in most practical scenarios given the comparable performance across the 3 modelling cases considered in this study. We recommend that modelling case 2 would be particularly advantageous in situations of sparse IPD, where one would like to shrink new coefficient estimates towards the existing CPMs to avoid overfitting.^11^

CPM aggregation is a relatively new concept, and so there remain areas for further research. For instance, datasets across populations frequently collect or record different variables, potentially meaning a variable included in an existing CPM is not available in the new IPD; this would restrict the ability to calculate the linear predictor of the existing model. The effect of systematically missing covariates on model aggregation is currently unknown, with the current analysis assuming the IPD records all variables. Previous work has indicated that multiple imputation with fixed and random effects is advantageous for imputing systematically missing covariates in multiple IPD meta‐analysis, but it is unclear how this would translate into model aggregation where only 1 IPD is available.^40^, ^41^ One would at least require information on the covariance structure of the previous data that the existing models were derived on. Practically, a common approach in the case of clinically recorded risk factors is to treat missing covariate data as null risk, but the bias induced on the calculated linear predictors and the corresponding effect on model aggregation is unknown. Secondly, all model aggregation techniques are susceptible to collinearity issues because each existing model aims to predict the same outcome, and each includes a very similar subset of covariates. Classically, the weights in stacked regression are restricted to be non‐negative to aid interpretation and avoid negative coefficients caused by including multiple collinear linear predictors^16^; however, the full impact of collinearity on this methodology is unknown.^12^ To this end, alternative model aggregation approaches that use principal component analysis or partial least squares might be beneficial.^13^ Speculatively, the use of penalisation within the hybrid method could mitigate the effects of modelling across a potentially large number of collinear existing CPMs; we recommend a detailed investigation into the effects of collinearity on model aggregation, and the potential of the hybrid method to overcome its effects. Finally, one could exploit and incorporate the stability of coefficients of individual covariates between existing models into model aggregation. For instance, covariates that have stable coefficient estimates across existing models/populations should arguably provide more information into the aggregate model than highly heterogeneous estimates. We recommend further work in each of the above areas.

While the strength of this work is in the evaluation of the proposed method in a real‐world clinical example and systematic simulation studies, there remain some important limitations. Firstly, the effects of publication bias or failing to select all existing CPMs were not analysed here. While the former would lead to an overestimation of aggregate regression coefficients, the latter could potentially inflate the variance because the aggregation would be based on an incomplete list of existing CPMs. Because the hybrid method is estimated using lasso regression (Equation (4)), it could be used to select from a potentially substantial number of existing CPMs identified by a systematic review of the literature. Secondly, we only applied the hybrid method to one clinical example, and so the results will need confirmation in other situations to assess generalisability. Finally, this study considered the validation of all models in data samples derived from populations similar to those used for model development (ie, “true” internal validation). Although, external validation is required to assess the generalisability of a model across many populations, we aimed to focus on the situation of developing a CPM for a defined/local population. Arguably, by combining multiple CPMs—or, preferably, by directly utilising multiple IPD^19^, ^20^, ^21^—one would obtain a model that can be generalised across populations.

The main implication of this work is the potential to incorporate existing CPMs, new IPD, and prior clinical knowledge into the modelling strategy. Generally, this aims to avoid disregarding existing CPMs after transferring them to a new population of interest.^12^, ^13^ It is worth emphasising that adoption of the proposed hybrid method might lead to a situation where multiple CPMs are each developed based on a collection of previously published models (which may themselves have been derived using the hybrid method). Therefore, the existing CPMs used within the hybrid method should have each been derived appropriately (in terms of adequate sample size^13^ and statistical methodology^1^); this would be equally applicable to other model aggregation methods.^16^, ^17^ However, unlike stacked regression, the proposed hybrid method can revise the prognostic effects of individual covariates, and, therefore, might be more robust against poorly specified existing CPMs. Moreover, one should acknowledge that introducing a CPM within clinical practice could be regarded as an intervention, which will inevitably alter the underlying risk processes—a so‐called “prediction paradox.” Thus, one frequently observes CPMs drifting out of calibration through time.^42^ It is conceivable that the hybrid method could be used iteratively (based on previous versions of itself) to continuously adapt the model to the local population. Further work is required to explore this idea.

In conclusion, this study presents a novel method of incorporating IPD, existing CPMs and clinical prior knowledge into model aggregation and model updating techniques. Through utilising multiple existing CPMs, the hybrid method consistently outperformed updating any model individually and consistently gave highest predictive performance across IPD sample sizes. Importantly, the method allows the existing models to have heterogeneous risk factor sets, and facilitates selection from a (potentially large) pool of existing CPMs. Thus, by penalising new parameters, the proposed modelling strategy can help choose between utilising existing CPMs and developing a model de novo.

## DISCLOSURES

The authors have no conflict of interest.

## Supporting information


**Supporting Information A**: Mathematical details of the synthetic simulation study design and supplementary tables from the synthetic simulation study.
**Supporting Information B**: Supplementary tables for the TAVI application analysis.
**Synthetic simulation study R code**: The R code used to run the simulation study based on the synthetic data.
**Empirical simulation study R code**: The R code used to run the simulation study based on the UK TAVI registry.
