# Supplementary material for: A multiple‐model generalisation of updating clinical prediction models
Source: Stat Med. 2017 Dec 18;37(8):1343–58. doi: 10.1002/sim.7586 (PMC5873448; doi:10.1002/sim.7586)
Supplement: Supplementary file 1 — Supporting Information A: Mathematical details of the synthetic simulation study design and supplementary tables from the synthetic simulation study. Supporting Information B: Supplementary tables for the TAVI application analysis. Synthetic simulation study R code: The R code used to run the simulation study based on the synthetic data. Empirical simulation study R code: The R code used to run the simulation study based on the UK TAVI registry. [file SIM-37-1343-s001.zip › Supplementary Material A.docx]

Supporting information A: Synthetic Simulation Methods and Results

Glen P. Martin; Mamas A. Mamas; Niels Peek; Iain Buchan; Matthew Sperrin

# Supplementary Methods for the Synthetic Simulation Design

## Data-generating mechanism: covariate data

The data-generating mechanism followed that from previous studies ^1^. Let $\boldsymbol{X}_{N\times P}$ denote the $N\times P$ matrix of predictors, where *N* is the cumulative sample size across all the generated populations. We generated *P=*50 predictors within 10 clusters of serially correlated variables, so that each cluster included *K=*5 predictors. Within each cluster, the simulated predictors had correlation $\rho=0.75$, but were independent between clusters. Specifically, the process to generate the predictors ($p=1,\ldots,P$) was as follows:

1. If cluster $\kappa$ includes only continuous predictors then simulate *N* realisations of the predictors at the ‘start’ of the cluster as

$$\boldsymbol{X}_{p}\sim\mathrm{Normal}\left( 0,1 \right),$$

and simulate the remaining *K-1* correlated predictors as

$$\boldsymbol{X}_{p}\sim\rho\boldsymbol{X}_{p-1}+\sqrt{(1-\rho^{2})}\boldsymbol{\Psi}\boldsymbol{,}$$

where $\boldsymbol{\Psi}\sim\mathrm{Normal}\left( 0,1 \right)$.

1. Else, if cluster $\kappa$ includes only binary predictors, we generate them as latent Normal. Specifically, simulate *N* realisations of the predictors at the ‘start’ of each cluster as

$$\boldsymbol{X}_{p}\sim\mathrm{Normal}\left( 0,1 \right),$$

and simulate the remaining *K-1* correlated predictors as

$$\boldsymbol{X}_{p}\sim\left\{ \begin{matrix} \boldsymbol{X}_{p-1} with prob. \rho\\ \boldsymbol{\Psi} with prob. 1-\rho\end{matrix} \right.$$

where $\boldsymbol{\Psi}\sim\mathrm{Normal}\left( 0,1 \right)$. Then dichotomize each variable in the cluster to give a pre-defined cluster-specific event rate between 10% and 50%, which are values frequently reported in observational datasets.

1. Repeat steps 1 to 2 across all 1$0$ clusters.

## Data-generating mechanism: outcome data

A binary response, $Y_{i,j}$, for individual $i$ in population *j* were sampled from a population-specific generating logistic regression model with $P\left( Y_{i,j}=1 \right)=q_{i,j},$ where

$$\log\left( \frac{q_{i,j}}{1-q_{i,j}} \right)=\alpha_{0,j}+ \sum_{p=1}^{P=50} \alpha_{p,j}x_{i,j,p},$$

with intercept $\alpha_{0,j}$ and generating coefficients $\alpha_{1,j},\ldots,\alpha_{P,j}$. If $\bar{\boldsymbol{\alpha}}$ represents the vector of mean predictor effects across all populations, then the simulation mechanism in each population *j* and generating parameter $p=1,\ldots,50$ was

$$\alpha_{p,j}\sim\left\{ \begin{matrix} N(\bar{\alpha}_{p},\sigma^{2}) & \mathrm{if}p\equiv1 (mod K=5) \\ 0 & \mathrm{otherwise} \end{matrix} \right.$$

The $p\equiv1 (mod K=5)$ condition implies (without loss of generality) that in each population, all non-zero generating coefficients were those at the ‘start’ of each cluster. Further, such a simulation procedure induces between-population-heterogeneity by applying random variation to the mean predictor-effects ($\bar{\boldsymbol{\alpha}}$), which was controlled through the value of σ that was introduced in the main text. To represent coefficients frequently reported in published models, $\bar{\boldsymbol{\alpha}}$ was sampled in each simulation as follows:

$$\bar{\alpha}_{p}\sim\left\{ \begin{matrix} Uniform(0.80, 1.6) & if parameter p is binary \\ Uniform(0.08, 0.1) & if parameter p is continuous \end{matrix} \right.$$

In addition, baseline risk undoubtedly differs between populations and, as such, each intercept $\alpha_{0,j}$was selected to give an average pre-defined event rate of 25% plus random variation.

# Supplementary Tables

**Table A1:** Mean Square Error (empirical standard error) in the predicted risks from model revision, stacked regression, the hybrid models and ridge regression for the synthetic simulation study. Bold items indicate the minimum mean square error in each combination of IPD sample size and value of σ.

| **Model** | **IPD Sample Size** | **σ= 0.000** | **σ=0.125** | **σ= 0.250** | **σ= 0.375** | **σ= 0.500** | **σ= 0.750** |
| --- | --- | --- | --- | --- | --- | --- | --- |
| Model Revision* | 200 | 0.038 (0.008) | 0.039 (0.009) | 0.042 (0.009) | 0.045 (0.011) | 0.047 (0.012) | 0.051 (0.016) |
| Stacked regression | 200 | **0.031 (0.006)** | **0.033 (0.007)** | **0.035 (0.008)** | 0.040 (0.010) | 0.042 (0.012) | 0.050 (0.015) |
| Hybrid Case 1 | 200 | 0.032 (0.007) | **0.033 (0.007)** | **0.035 (0.008)** | **0.037 (0.010)** | **0.038 (0.011)** | **0.040 (0.014)** |
| Hybrid Case 2 | 200 | 0.032 (0.007) | 0.034 (0.007) | **0.035 (0.008)** | **0.037 (0.010)** | **0.038 (0.011)** | 0.041 (0.014) |
| Hybrid Case 3 | 200 | 0.035 (0.008) | 0.036 (0.008) | 0.037 (0.009) | 0.039 (0.010) | 0.040 (0.011) | 0.042 (0.014) |
| Ridge regression | 200 | 0.037 (0.006) | 0.037 (0.007) | 0.037 (0.008) | 0.039 (0.009) | 0.040 (0.010) | 0.042 (0.013) |
| Model Revision* | 300 | 0.036 (0.007) | 0.038 (0.008) | 0.040 (0.009) | 0.041 (0.010) | 0.044 (0.012) | 0.048 (0.016) |
| Stacked regression | 300 | **0.029 (0.006)** | **0.031 (0.007)** | 0.034 (0.008) | 0.037 (0.009) | 0.040 (0.011) | 0.048 (0.015) |
| Hybrid Case 1 | 300 | 0.030 (0.006) | **0.031 (0.007)** | **0.032 (0.008)** | **0.033 (0.009)** | **0.034 (0.010)** | **0.036 (0.013)** |
| Hybrid Case 2 | 300 | 0.030 (0.006) | **0.031 (0.007)** | **0.032 (0.007)** | 0.034 (0.009) | 0.035 (0.010) | 0.037 (0.013) |
| Hybrid Case 3 | 300 | 0.031 (0.007) | 0.032 (0.007) | 0.033 (0.008) | 0.034 (0.009) | 0.035 (0.011) | 0.037 (0.014) |
| Ridge regression | 300 | 0.033 (0.006) | 0.034 (0.007) | 0.034 (0.007) | 0.035 (0.008) | 0.036 (0.010) | 0.038 (0.013) |
| Model Revision* | 500 | 0.035 (0.007) | 0.036 (0.008) | 0.038 (0.009) | 0.040 (0.010) | 0.041 (0.012) | 0.046 (0.016) |
| Stacked regression | 500 | **0.028 (0.005)** | 0.029 (0.006) | 0.032 (0.008) | 0.035 (0.009) | 0.039 (0.011) | 0.047 (0.015) |
| Hybrid Case 1 | 500 | **0.028 (0.006)** | **0.028 (0.006)** | **0.029 (0.007)** | **0.030 (0.008)** | **0.031 (0.010)** | **0.033 (0.013)** |
| Hybrid Case 2 | 500 | **0.028 (0.006)** | 0.029 (0.006) | 0.030 (0.007) | 0.031 (0.008) | 0.032 (0.010) | 0.034 (0.013) |
| Hybrid Case 3 | 500 | 0.029 (0.006) | 0.029 (0.006) | 0.030 (0.007) | 0.031 (0.009) | 0.032 (0.010) | **0.033 (0.013)** |
| Ridge regression | 500 | 0.031 (0.005) | 0.030 (0.006) | 0.031 (0.007) | 0.032 (0.008) | 0.033 (0.010) | 0.035 (0.012) |
| Model Revision* | 1000 | 0.034 (0.007) | 0.035 (0.007) | 0.036 (0.008) | 0.037 (0.010) | 0.039 (0.011) | 0.043 (0.015) |
| Stacked regression | 1000 | 0.027 (0.005) | 0.028 (0.006) | 0.031 (0.007) | 0.034 (0.009) | 0.038 (0.011) | 0.046 (0.014) |
| Hybrid Case 1 | 1000 | **0.026 (0.006)** | **0.027 (0.006)** | **0.027 (0.007)** | **0.028 (0.008)** | **0.028 (0.010)** | **0.030 (0.013)** |
| Hybrid Case 2 | 1000 | **0.026 (0.006)** | **0.027 (0.006)** | 0.028 (0.007) | **0.028 (0.008)** | 0.029 (0.010) | 0.031 (0.013) |
| Hybrid Case 3 | 1000 | **0.026 (0.006)** | **0.027 (0.006)** | **0.027 (0.007)** | **0.028 (0.008)** | **0.028 (0.010)** | **0.030 (0.013)** |
| Ridge regression | 1000 | 0.028 (0.006) | 0.028 (0.006) | 0.028 (0.007) | 0.029 (0.008) | 0.029 (0.010) | 0.031 (0.013) |
| Model Revision* | 2000 | 0.034 (0.007) | 0.035 (0.007) | 0.035 (0.008) | 0.036 (0.010) | 0.038 (0.012) | 0.043 (0.015) |
| Stacked regression | 2000 | 0.026 (0.005) | 0.028 (0.006) | 0.030 (0.007) | 0.034 (0.009) | 0.038 (0.011) | 0.045 (0.014) |
| Hybrid Case 1 | 2000 | **0.025 (0.005)** | **0.025 (0.006)** | **0.026 (0.007)** | **0.026 (0.008)** | **0.027 (0.010)** | **0.029 (0.013)** |
| Hybrid Case 2 | 2000 | **0.025 (0.005)** | 0.026 (0.006) | **0.026 (0.007)** | 0.027 (0.008) | **0.027 (0.010)** | **0.029 (0.013)** |
| Hybrid Case 3 | 2000 | **0.025 (0.005)** | 0.026 (0.006) | **0.026 (0.007)** | 0.027 (0.008) | **0.027 (0.010)** | **0.029 (0.013)** |
| Ridge regression | 2000 | 0.026 (0.005) | 0.026 (0.006) | **0.026 (0.007)** | 0.027 (0.008) | 0.028 (0.010) | 0.030 (0.013) |
| Model Revision* | 5000 | 0.033 (0.007) | 0.033 (0.007) | 0.034 (0.009) | 0.036 (0.010) | 0.037 (0.012) | 0.041 (0.015) |
| Stacked regression | 5000 | 0.026 (0.005) | 0.027 (0.006) | 0.030 (0.007) | 0.034 (0.009) | 0.037 (0.011) | 0.045 (0.014) |
| Hybrid Case 1 | 5000 | **0.024 (0.005)** | **0.025 (0.006)** | **0.025 (0.007)** | **0.025 (0.009)** | **0.026 (0.010)** | **0.027 (0.013)** |
| Hybrid Case 2 | 5000 | **0.024 (0.005)** | **0.025 (0.006)** | **0.025 (0.007)** | 0.026 (0.009) | **0.026 (0.010)** | **0.027 (0.013)** |
| Hybrid Case 3 | 5000 | **0.024 (0.005)** | **0.025 (0.006)** | **0.025 (0.007)** | **0.025 (0.009)** | **0.026 (0.010)** | **0.027 (0.013)** |
| Ridge regression | 5000 | **0.024 (0.005)** | **0.025 (0.006)** | **0.025 (0.007)** | 0.026 (0.008) | **0.026 (0.010)** | 0.028 (0.012) |

**: results of model revision from one of the simulated existing CPMs, with results being quantitatively similar across all five simulated existing CPMs*

**Table A2:** Additional AUC (standard error) results for model revision, stacked regression, the hybrid models and ridge regression from the synthetic simulation study. Bold items indicate the maximum AUC in each combination of IPD sample size and value of σ.

| **Model** | **IPD Sample Size** | **σ= 0.000** | **σ=0.125** | **σ= 0.250** | **σ= 0.375** | **σ= 0.500** | **σ= 0.750** |
| --- | --- | --- | --- | --- | --- | --- | --- |
| Model Revision* | 300 | 0.682 (0.009) | 0.678 (0.009) | 0.681 (0.009) | 0.691 (0.009) | 0.704 (0.008) | 0.731 (0.008) |
| Stacked regression | 300 | **0.717 (0.008)** | 0.715 (0.008) | 0.713 (0.008) | 0.716 (0.008) | 0.720 (0.008) | 0.733 (0.008) |
| Hybrid Case 1 | 300 | 0.716 (0.008) | **0.716 (0.008)** | **0.722 (0.008)** | **0.732 (0.008)** | **0.747 (0.008)** | **0.780 (0.007)** |
| Hybrid Case 2 | 300 | 0.716 (0.008) | 0.715 (0.008) | 0.721 (0.008) | 0.731 (0.008) | 0.744 (0.008) | 0.776 (0.007) |
| Hybrid Case 3 | 300 | 0.709 (0.008) | 0.709 (0.008) | 0.716 (0.008) | 0.727 (0.008) | 0.742 (0.008) | 0.776 (0.007) |
| Ridge regression | 300 | 0.697 (0.008) | 0.700 (0.008) | 0.710 (0.008) | 0.723 (0.008) | 0.738 (0.008) | 0.772 (0.007) |
| Model Revision* | 2000 | 0.687 (0.009) | 0.688 (0.009) | 0.698 (0.008) | 0.712 (0.008) | 0.722 (0.008) | 0.75 (0.008) |
| Stacked regression | 2000 | 0.727 (0.008) | 0.724 (0.008) | 0.723 (0.008) | 0.725 (0.008) | 0.726 (0.008) | 0.743 (0.008) |
| Hybrid Case 1 | 2000 | **0.731 (0.008)** | **0.734 (0.008)** | **0.744 (0.008)** | **0.757 (0.008)** | **0.770 (0.008)** | **0.801 (0.007)** |
| Hybrid Case 2 | 2000 | **0.731 (0.008)** | 0.733 (0.008) | 0.743 (0.008) | 0.756 (0.008) | 0.769 (0.008) | 0.800 (0.007) |
| Hybrid Case 3 | 2000 | **0.731 (0.008)** | **0.734 (0.008)** | 0.743 (0.008) | **0.757 (0.008)** | **0.770 (0.008)** | 0.800 (0.007) |
| Ridge regression | 2000 | 0.728 (0.008) | 0.731 (0.008) | 0.741 (0.008) | 0.754 (0.008) | 0.767 (0.008) | 0.798 (0.007) |

# Supplementary References

1. Martin GP, Mamas MA, Peek N, Buchan I, Sperrin M. Clinical prediction in defined populations: a simulation study investigating when and how to aggregate existing models. *BMC Med. Res. Methodol.* 2017;17(1):1. doi:10.1186/s12874-016-0277-1.
