# Supplementary material for: A multiple‐model generalisation of updating clinical prediction models
Source: Stat Med. 2017 Dec 18;37(8):1343–58. doi: 10.1002/sim.7586 (PMC5873448; doi:10.1002/sim.7586)
Supplement: Supplementary file 1 — Supporting Information A: Mathematical details of the synthetic simulation study design and supplementary tables from the synthetic simulation study. Supporting Information B: Supplementary tables for the TAVI application analysis. Synthetic simulation study R code: The R code used to run the simulation study based on the synthetic data. Empirical simulation study R code: The R code used to run the simulation study based on the UK TAVI registry. [file SIM-37-1343-s001.zip › Supplementary Material B.docx]

Supporting Information B: Supplementary Tables for the TAVI Application Analysis

Glen P. Martin; Mamas A. Mamas; Niels Peek; Iain Buchan; Matthew Sperrin

**Table B1:** Parameter estimates from model revision, stacked regression (SR), hybrid and re-development modelling approaches in the TAVI cohort.

| **Variable** | **German AV Revision** | **FRANCE-2 Revision** | **OBSERVANT Revision** | **ACC Revision** | **SR** | **Hybrid Case 1** | **Hybrid Case 2** | **Hybrid Case 3** | **AIC** | **Ridge** |
| --- | --- | --- | --- | --- | --- | --- | --- | --- | --- | --- |
| Intercept | -4.135 | -3.605 | -3.378 | -4.000 | -4.421 | -4.061 | -4.444 | -4.086 | -3.250 | -3.773 |
| Age 66-70 | 0.164 | - | - | - | 0.107 | 0.077 | 0.107 | 0.064 | - | -0.060 |
| Age 71-75 | 0.322 | - | - | - | 0.211 | 0.126 | 0.211 | 0.126 | -0.317 | -0.144 |
| Age 76-80 | 0.459 | - | - | - | 0.300 | 0.229 | 0.300 | 0.180 | - | 0.044 |
| Age 81-85 | 0.633 | - | - | - | 0.413 | 0.298 | 0.414 | 0.248 | - | -0.002 |
| Age >85 | 0.835 | - | - | - | 0.545 | 0.393 | 0.546 | 0.327 | - | 0.042 |
| Age >90 | - | 0.248 | - | - | 0.136 | 0.102 | 0.088 | 0.030 | 0.407 | 0.232 |
| Age per 5-years | - | - | - | 0.066 | 0.035 | 0.020 | 0.029 | 0.023 | - | 0.021 |
| Female | 0.127 | - | - | - | 0.083 | 0.071 | 0.083 | 0.050 | - | 0.089 |
| BMI <22 | 0.605 | - | - | - | 0.083 | 0.420 | 0.367 | 0.334 | 0.646 | 0.372 |
| BMI <18.5 | - | 0.483 | - | - | 0.265 | 0.137 | 0.185 | 0.073 | - | 0.270 |
| BMI 18.5-29.9 | - | -0.018 | - | - | 0.132 | 0.039 | 0.076 | 0.020 | - | -0.018 |
| BMI >35 | 0.140 | - | - | - | 0.091 | 0.111 | 0.104 | 0.067 | 0.326 | 0.153 |
| NYHA Class IV | 0.189 | 0.342 | 0.266 | 0.120 | 0.381 | 0.214 | 0.337 | 0.147 | - | 0.120 |
| MI within 3 weeks | 0.293 | - | - | - | 0.191 | 0.138 | 0.192 | 0.115 | - | 0.139 |
| Critical Pre-op | 0.873 | 0.513 | 0.945 | - | 0.443 | 0.467 | 0.383 | 0.138 | 2.392 | 0.546 |
| Pulmonary Hypertension | 0.141 | 0.218 | 0.266 | - | 0.218 | 0.140 | 0.214 | 0.075 | 0.289 | 0.146 |
| No Sinus Rhythm | 0.122 | - | - | - | 0.080 | 0.087 | 0.081 | 0.049 | 0.223 | 0.136 |
| LVEF 30-50% | 0.101 | - | - | - | 0.066 | 0.048 | 0.066 | 0.039 | - | 0.043 |
| LVEF <30% | 0.202 | - | - | - | 0.132 | 0.108 | 0.132 | 0.079 | - | 0.126 |
| LVEF <40% | - | - | 0.200 | - | 0.005 | 0.028 | 0.038 | 0.000 | - | 0.083 |
| Prior Cardiac Surgery | 0.109 | - | - | - | 0.071 | 0.030 | 0.070 | 0.041 | - | -0.069 |
| Arterial vessel disease | 0.508 | - | - | - | 0.083 | 0.127 | 0.102 | 0.068 | 0.247 | 0.200 |
| COPD | 0.113 | 0.295 | - | 0.275 | 0.383 | 0.225 | 0.290 | 0.166 | 0.280 | 0.156 |
| Dialysis | 0.413 | 0.625 | - | 0.635 | 0.953 | 0.492 | 0.750 | 0.439 | - | 0.161 |
| Emergency | 1.345 | - | - | - | 0.245 | 0.291 | 0.340 | 0.241 | - | 0.504 |
| Non-TF Access | - | - | - | 0.751 | 0.195 | 0.164 | 0.159 | 0.126 | - | 0.286 |
| TA Access | - | 0.951 | - | - | 0.226 | 0.596 | 0.594 | 0.499 | 0.886 | 0.432 |
| Other Access | - | 0.460 | - | - | 0.252 | 0.073 | 0.148 | 0.042 | 0.288 | -0.031 |
| eGFR <45$mL/min$ | - | - | 0.399 | - | 0.010 | 0.050 | 0.076 | 0.000 | - | 0.130 |
| eGFR per 5-units | - | - | - | -0.037 | -0.020 | -0.016 | -0.016 | -0.013 | -0.043 | -0.016 |
| Diabetes | - | - | -0.234 | - | 0.007 | -0.005 | 0.041 | -0.010 | - | -0.086 |
| Prior BAV | - | - | 0.200 | - | 0.005 | 0.126 | 0.079 | 0.041 | 0.327 | 0.199 |
| Acuity Category 2 | - | - | - | 0.243 | 0.131 | 0.133 | 0.108 | 0.086 | 0.403 | 0.216 |
| Acuity Category 3 | - | - | - | 0.535 | 0.287 | 0.007 | 0.235 | 0.186 | -1.852 | -0.032 |
| Acuity Category 4 | - | - | - | 1.515 | 0.349 | 0.584 | 0.451 | 0.392 | - | 0.583 |

*ACC: American College of Cardiology model, BAV: Balloon Aortic Valvuloplasty, BMI: Body Mass Index, COPD: Chronic Obstructive Pulmonary Disease, eGFR: Estimated glomerular filtration rate, German Aortic Valve model, LVEF: Left Ventricular Ejection Fraction, MI: Myocardial Infarction, TF: Transfemoral, TA: Transapical.*

**Table B2:** Parameter estimates from model extension, stacked regression (SR), hybrid and re-development modelling approaches in the TAVI cohort for the sensitivity analysis that considered the addition of frailty (KATZ and CSHA scores) in the models.

| **Variable** | **German AV Extension** | **FRANCE-2 Extension** | **OBSERVANT Extension** | **ACC Extension** | **SR** | **Hybrid Case 1** | **Hybrid Case 2** | **Hybrid Case 3** | **AIC** | **Ridge** |
| --- | --- | --- | --- | --- | --- | --- | --- | --- | --- | --- |
| Intercept | -4.313 | -3.946 | -3.734 | -4.229 | -4.421 | -4.172 | -4.528 | -4.227 | -3.607 | -3.879 |
| Age 66-70 | 0.132 | - | - | - | 0.107 | 0.062 | 0.090 | 0.036 | - | -0.054 |
| Age 71-75 | 0.261 | - | - | - | 0.211 | 0.098 | 0.177 | 0.071 |  | -0.129 |
| Age 76-80 | 0.371 | - | - | - | 0.300 | 0.187 | 0.252 | 0.101 | - | 0.044 |
| Age 81-85 | 0.512 | - | - | - | 0.413 | 0.239 | 0.348 | 0.139 | - | -0.010 |
| Age >85 | 0.676 | - | - | - | 0.545 | 0.315 | 0.459 | 0.184 | - | 0.032 |
| Age >90 | - | 0.199 | - | - | 0.136 | 0.108 | 0.087 | 0.031 | 0.404 | 0.238 |
| Age per 5-years | - | - | - | 0.053 | 0.035 | 0.018 | 0.026 | 0.017 | - | 0.014 |
| Female | 0.103 | - | - | - | 0.083 | 0.056 | 0.070 | 0.028 | - | 0.071 |
| BMI <22 | 0.551 | - | - | - | 0.083 | 0.394 | 0.369 | 0.327 | 0.594 | 0.359 |
| BMI <18.5 | - | 0.934 | - | - | 0.265 | 0.134 | 0.170 | 0.060 | - | 0.252 |
| BMI 18.5-29.9 | - | 0.195 | - | - | 0.132 | 0.027 | 0.066 | 0.011 | - | -0.021 |
| BMI >35 | 0.113 | - | - | - | 0.091 | 0.095 | 0.098 | 0.052 | 0.331 | 0.165 |
| NYHA Class IV | 0.153 | 0.275 | 0.222 | 0.097 | 0.381 | 0.176 | 0.291 | 0.090 | - | 0.080 |
| MI within 3 weeks | 0.237 | - | - | - | 0.191 | 0.128 | 0.161 | 0.065 | - | 0.161 |
| Critical Pre-op | 0.817 | 0.413 | 0.857 | - | 0.443 | 0.459 | 0.328 | 0.076 | 2.161 | 0.502 |
| Pulmonary Hypertension | 0.114 | 0.176 | 0.222 | - | 0.218 | 0.110 | 0.184 | 0.041 | 0.289 | 0.154 |
| No Sinus Rhythm | 0.099 | - | - | - | 0.080 | 0.072 | 0.072 | 0.032 | 0.200 | 0.128 |
| LVEF 30-50% | 0.081 | - | - | - | 0.066 | 0.038 | 0.055 | 0.022 | - | 0.035 |
| LVEF <30% | 0.164 | - | - | - | 0.132 | 0.087 | 0.111 | 0.045 | - | 0.101 |
| LVEF <40% | - | - | 0.167 | - | 0.005 | 0.025 | 0.035 | 0.000 | - | 0.067 |
| Prior Cardiac Surgery | 0.088 | - | - | - | 0.071 | 0.024 | 0.060 | 0.024 | - | -0.058 |
| Arterial vessel disease | 0.451 | - | - | - | 0.083 | 0.107 | 0.095 | 0.053 | 0.235 | 0.184 |
| COPD | 0.091 | 0.237 | - | 0.222 | 0.383 | 0.199 | 0.251 | 0.112 | 0.251 | 0.151 |
| Dialysis | 0.335 | 0.503 | - | 0.511 | 0.953 | 0.424 | 0.649 | 0.289 | - | 0.153 |
| Emergency | 1.289 | - | - | - | 0.245 | 0.239 | 0.292 | 0.168 | - | 0.476 |
| Non-TF Access | - | - | - | 0.701 | 0.195 | 0.135 | 0.143 | 0.096 | - | 0.276 |
| TA Access | - | 0.922 | - | - | 0.226 | 0.622 | 0.624 | 0.531 | 0.837 | 0.437 |
| Other Access | - | 0.370 | - | - | 0.252 | 0.066 | 0.126 | 0.022 | - | -0.059 |
| eGFR <45$mL/min$ | - | - | 0.333 | - | 0.010 | 0.038 | 0.070 | 0.000 | - | 0.122 |
| eGFR per 5-units | - | - | - | -0.030 | -0.020 | -0.014 | -0.015 | -0.010 | -0.039 | -0.015 |
| Diabetes | - | - | -0.240 | - | 0.007 | -0.014 | 0.032 | -0.015 | - | -0.096 |
| Prior BAV | - | - | 0.167 | - | 0.005 | 0.105 | 0.091 | 0.056 | 0.281 | 0.184 |
| Acuity Category 2* | - | - | - | 0.196 | 0.131 | 0.112 | 0.096 | 0.065 | 0.307 | 0.192 |
| Acuity Category 3* | - | - | - | 0.431 | 0.287 | -0.064 | 0.205 | 0.136 | -1.763 | -0.075 |
| Acuity Category 4* | - | - | - | 1.439 | 0.349 | 0.603 | 0.560 | 0.476 | - | 0.580 |
| KATZ<6 Frailty | 0.528 | 0.543 | 0.516 | 0.522 | - | 0.339 | 0.323 | 0.323 | 0.475 | 0.298 |
| CSHA Frailty | 0.459 | 0.473 | 0.529 | 0.446 | - | 0.217 | 0.173 | 0.173 | 0.390 | 0.234 |

**Defined as a composite of procedure urgency, pre-procedure shock, inotropes, mechanical assist device, or cardiac arrest* [1]*. ACC: American College of Cardiology model, BAV: Balloon Aortic Valvuloplasty, BMI: Body Mass Index, COPD: Chronic Obstructive Pulmonary Disease, CSHA: Canadian Study of Health and Aging,, eGFR: Estimated glomerular filtration rate, German Aortic Valve model, LVEF: Left Ventricular Ejection Fraction, MI: Myocardial Infarction, TF: Transfemoral, TA: Transapical.*

# Supplementary References

1. Edwards FH, Cohen DJ, O’Brien SM, Peterson ED, Mack MJ, Shahian DM, Grover FL, Tuzcu EM, Thourani VH, Carroll J, Brennan JM, Brindis RG, Rumsfeld J, Holmes DR. Development and Validation of a Risk Prediction Model for In-Hospital Mortality After Transcatheter Aortic Valve Replacement. JAMA Cardiol. 2016;1:46.
